# Fingolimod Alleviates Neuroinflammation and Blood–Brain Barrier Disruption in Epilepsy by Upregulating S1PR5 and Inhibiting the Rho/ROCK/NF‐κB Pathway

**DOI:** 10.1002/cns.71212

**Published:** 2026-10-14

**Authors:** Chen Zhang, Yanli Duan, Mengmeng Ma, Lin Xu, Wei‐Na Jin, Zhuoran Zhang

**Affiliations:** ^1^ Department of Neurology Tianjin Medical University General Hospital Tianjin China; ^2^ Department of Clinical Laboratory Zhaotong Hospital Affiliated to Kunming Medical University Zhaotong Yunnan Province China; ^3^ China National Clinical Research Center for Neurological Diseases Beijing Tiantan Hospital, Capital Medical University Beijing China

**Keywords:** epilepsy, Fingolimod, rho/ROCK/NF‐κB, S1PR5

## Abstract

**Objective:**

This study aimed to clarify the role of sphingosine‐1‐phosphate receptor 5 (S1PR5) in epileptic pathogenesis and the mechanism by which fingolimod exerts anti‐epileptic effects, with a focus on neuroinflammation and blood–brain barrier (BBB) disruption.

**Methods:**

Multi‐omics integration and machine learning were used to identify S1PR5 as a key candidate gene. Molecular docking was performed to verify the binding affinity between fingolimod and S1PR5. AAV‐mediated S1PR5 overexpression was conducted in mouse hippocampi before kainic acid (KA) administration. KA‐induced epileptic mice were randomly divided into control, model, and fingolimod intervention groups. Behavioral tests, qRT‐PCR, Western blot, immunofluorescence, ELISA, and Evans Blue assay were used for evaluations.

**Results:**

S1PR5 expression was significantly downregulated in both epileptic patients and mouse hippocampi. Fingolimod bound stably to S1PR5 in silico. S1PR5 overexpression attenuated neuronal loss, microglial activation, and epileptic seizures in KA‐treated mice, confirming that S1PR5 exerts protective effects in epilepsy. Fingolimod intervention upregulated S1PR5 expression, inhibited abnormal activation of the Rho/ROCK/NF‐κB pathway, restored tight junction proteins (Claudin‐5 and ZO‐1), reduced BBB leakage, and lowered proinflammatory cytokines (TNF‐α, IL‐1β, IL‐6). These changes were accompanied by alleviated seizure severity and frequency.

**Conclusion:**

S1PR5 serves as a protective regulator in epilepsy. Fingolimod exerts anti‐epileptic effects by upregulating S1PR5, thereby inhibiting the Rho/ROCK/NF‐κB pathway, reducing neuroinflammation, and preserving BBB integrity. These findings support S1PR5 as a promising target and provide a mechanistic basis for repurposing fingolimod in epilepsy therapy.

## Graphical abstract

Fingolimod alleviates epilepsy by restoring S1PR5 expression and suppressing the Rho/ROCK/NF‐κB pathway, thereby reducing neuroinflammation, preserving blood–brain barrier integrity, and attenuating seizure activity.

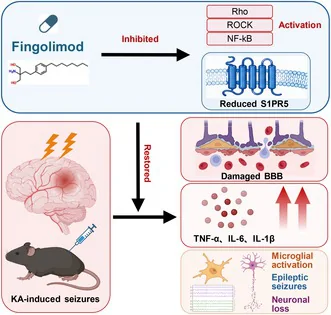

## 1 Introduction

Epilepsy is a disabling neurological disorder that markedly compromises patients' quality of life [1]. Although various antiepileptic drugs are available, most only control seizures symptomatically, and nearly one‐third of patients remain drug‐resistant. This limited efficacy is largely attributable to the inability of existing therapies to reverse the underlying pathological mechanisms of epilepsy [2]. Therefore, elucidating the molecular mechanisms of epileptogenesis and identifying novel therapeutic targets remain urgent priorities in neuroscience research.

Traditionally, epilepsy research has focused on an imbalance between neuronal excitation and inhibition [3]. However, emerging evidence indicates that neuroinflammation plays a pivotal role in epileptogenesis [4]. Seizures activate glial cells and promote the release of proinflammatory cytokines, such as TNF‐α and IL‐1β, thereby increasing neuronal excitability and compromising blood–brain barrier (BBB) integrity [5, 6]. As a critical structure maintaining the homeostasis of the intracerebral microenvironment, BBB dysfunction is increasingly recognized as an important factor in the progression of epilepsy [7]. Neuroinflammation drives BBB disruption—degrading tight junction proteins, impairing endothelial function, and increasing vascular permeability. This, in turn, facilitates the infiltration of peripheral toxins and immune cells into the brain, further aggravating neuroinflammation and neuronal damage and ultimately forming a “neuroinflammation‐BBB breakdown‐seizure” vicious cycle. This process may also impede drug penetration into the brain, contributing to treatment resistance, which remains a major clinical challenge [8, 9].

Within the complex regulatory network of neuroinflammation and BBB disruption, the Rho/ROCK signaling pathway has attracted considerable attention as a critical hub linking upstream inflammatory stimuli to downstream pathological change [10, 11, 12]. This pathway mediates cytoskeletal remodeling, promotes the release of inflammatory factors, and damages endothelial tight junctions. Meanwhile, NF‐κB is also activated in epilepsy to drive inflammatory amplification [13, 14]. Therefore, targeting the Rho/ROCK/NF‐κB axis may represent a promising approach to ameliorate neuroinflammation and BBB injury in epilepsy.

Sphingosine‐1‐phosphate receptor 5 (S1PR5), a member of the sphingosine‐1‐phosphate receptor family, is expressed in the central nervous system and participates in neuronal signal transduction and the maintenance of BBB homeostasis [15, 16]. Previous studies have shown that S1PR5 protects brain endothelial cells by suppressing NF‐κB and inflammatory mediators [10]. Our bioinformatics analysis revealed that S1PR5 expression was reduced in epilepsy and its associated pathways were closely linked to the Rho/ROCK signaling pathway. Molecular docking further demonstrated a high binding affinity between Fingolimod and S1PR5. However, whether Fingolimod targets S1PR5 to regulate Rho/ROCK/NF‐κB and thereby modify epileptic progression remains unclear.

Accordingly, the study integrated machine learning, network pharmacology, and molecular docking to identify key genes and signaling pathways involved in epilepsy, with significant enrichment of the Rho/ROCK pathway. We further investigated whether Fingolimod could alleviate neuroinflammation and BBB injury through regulation of the S1PR5/Rho/ROCK/NF‐κB axis in cellular and animal models.

## 2 Materials and Methods

### 2.1 Data Source

The epilepsy‐related dataset GSE143272 was downloaded from the Gene Expression Omnibus (GEO) database (https://www.ncbi.nlm.nih.gov/geo/), which contains 142 blood samples. A total of 63 samples with high intra‐group consistency were selected for analysis, including 32 epilepsy patients and 31 healthy controls, with the sequencing type being microarray sequencing.

### 2.2 Screening of Differentially Expressed Genes (DEGs)

The R package ‘limma’ (version 3.60.4) was used for inter‐group differential expression analysis. DEGs were identified with the screening criteria of *p*‐value < 0.05 and |log_2_ fold change (FC)| > 1. Volcano plots and heatmaps were used to visualize the results.

### 2.3 Functional Enrichment Analysis

The R package ‘clusterProfiler’ (version 4.12.6) was employed to perform Gene Ontology (GO, including cellular component, molecular function, and biological process) and Kyoto Encyclopedia of Genes and Genomes (KEGG) pathway enrichment analyses for DEGs (*p* < 0.05). Gene Set Enrichment Analysis (GSEA) was conducted using the GSEA function in R, with reference to the C2 (KEGG gene sets) from the Molecular Signatures Database (MSigDB).

### 2.4 Weighted Gene co‐Expression Network Analysis (WGCNA)

A gene co‐expression network was constructed using the R package ‘WGCNA’ (version 1.73). The soft‐thresholding power was set to 8, and the module merging threshold was set at 0.25. The correlation between modules and epilepsy phenotypes was calculated, and the module most relevant to the disease was screened. The intersection of genes in this module with DEGs was taken to obtain key differentially co‐expressed genes.

### 2.5 Protein–Protein Interaction (PPI) Network Construction and Core Gene Screening

Key differentially co‐expressed genes were uploaded to the STRING database to construct a PPI network (confidence score ≥ 0.7). The CytoNCA plugin in Cytoscape software (version 3.10.3) was used to screen core node genes.

### 2.6 Machine Learning for Feature Gene Screening

The R packages ‘glmnet’ (version 4.1–8; LASSO regression), ‘randomForest’ (random forest), and ‘e1071’ (support vector machine, SVM) were used respectively to screen feature genes. For random forest, the number of decision trees was set to 500 (ntree = 500), and the number of variables randomly sampled as candidates at each split (mtry) was set to sqrt(p), where p is the total number of features. For SVM, a radial basis function (RBF) kernel was applied. The intersection of genes identified by the three methods was taken as core feature genes. The R package ’pROC’ (version 1.18.5) was used to plot Receiver Operating Characteristic (ROC) curves, and the Area Under the Curve (AUC) value was used to evaluate the diagnostic efficacy of the genes.

### 2.7 Target Gene Regulatory Network and Molecular Docking

miRNAs interacting with S1PR5 were predicted from the miRanda, miRDB, and TargetScan databases (the intersection of the three was taken). Interacting lncRNAs were obtained from the SpongeScan database to construct a competitive endogenous RNA (ceRNA) network. The R package ‘RcisTarget’ (version 1.26.0) was used to predict potential transcription factors of S1PR5. Drugs binding to S1PR5 were predicted through the Enrichr platform. Protein and drug structures were retrieved from the Protein Data Bank (PDB; https://www.rcsb.org/) and PubChem (https://pubchem.ncbi.nlm.nih.gov/) databases, and molecular docking was performed using AutoDock 1.2.0 software.

### 2.8 Experimental Animals

Specific pathogen‐free (SPF) grade male C57BL/6 mice (6–8 weeks old, weighing 20–22 g) were purchased from the SiPeiFu Laboratory Animal Center. Mice were housed in an environment with constant temperature (22°C ± 2°C), constant humidity (45%–60%), and a 12 h light/12 h dark cycle, with free access to standard feed and sterile water. All animal experiments were approved by the Tianjin Medical University General Hospital Animal Ethics Committee (approval no.: IRB2025‐DWFL‐662) and strictly conducted in accordance with the National Institutes of Health (NIH) *Guide for the Care and Use of Laboratory Animals*.

### 2.9 Main Reagents and Antibodies

Kainic acid (KA; purity ≥ 98%) was purchased from Sigma‐Aldrich (St. Louis, MO, USA). Fingolimod (purity ≥ 99%) was purchased from MedChemExpress (USA).

Primary antibodies included rabbit anti‐S1PR5 (Cat. No. PA5‐98877), mouse anti‐Claudin‐5 (Cat. No. 35–2500), and rabbit anti‐ZO‐1 (Cat. No. 617300), which were purchased from Thermo Fisher Scientific (USA), as well as rabbit anti‐RhoA (Cat. No. 2117 T), rabbit anti‐ROCK1 (Cat. No. 28999 T), rabbit anti‐ROCK2 (Cat. No. 47012 T), rabbit anti‐phospho‐NF‐κB p65 (Ser536) (Cat. No. 3033 T), rabbit anti‐NF‐κB p65 (Cat. No. 8242 T), and rabbit anti‐GAPDH (Cat. No. 2118 T), which were purchased from Cell Signaling Technology (USA).

Secondary antibodies included horseradish peroxidase (HRP)‐conjugated goat anti‐rabbit IgG (Cat. No. 31460), Alexa Fluor 488‐conjugated donkey anti‐mouse IgG (Cat. No. A21202), and Alexa Fluor 488‐conjugated donkey anti‐rabbit IgG (Cat. No. A21206), all purchased from Thermo Fisher Scientific. Enzyme‐linked immunosorbent assay (ELISA) kits for mouse tumor necrosis factor‐α (TNF‐α; Cat. No. SU‐BN20582), interleukin‐1β (IL‐1β; Cat. No. SU‐BN20174), interleukin‐6 (IL‐6; Cat. No. SU‐BN20188) and interleukin‐10 (IL‐6; Cat. No. SU‐BN20162) were purchased from Czkewei (China), S1PR5 (EM1342) were purchased from FineTest (China).

The RNA extraction kit (RNeasy Mini Kit, Cat. No. ER501), reverse transcription kit (PrimeScript RT Reagent Kit with gDNA Eraser, Cat. No. AT311‐02), and qRT‐PCR kit (TB Green Premix Ex Taq II, Cat. No. AQ601) were purchased from TransGen Biotech (China). Evans Blue (EB; Cat. No. E2129) was purchased from Sigma‐Aldrich (USA).

### 2.10 Experimental Grouping, Model Establishment, and Drug Intervention

Mice were randomly assigned to three groups (*n* = 4–5 per group): control, KA, and KA + Fingolimod. Epilepsy was induced in the KA and KA + Fingolimod groups by intraperitoneal injection of KA (25 mg/kg, dissolved in PBS). Following KA administration, mice were observed for 30–60 min, and seizure severity was assessed using the Racine scale. A Racine score of ≥ 3 was defined as successful model induction, and the seizure score of each mouse was recorded. Mice in the KA + Fingolimod group received Fingolimod (6 mg/kg, intraperitoneally) within 1 h after the first seizure onset and once every 24 h thereafter. Control mice received an equal volume of normal saline on the same schedule. After 7 days, all mice were sacrificed and brain tissues were collected for subsequent experiments.

### 2.11 Sample Collection

At 24 h or 7 days after KA injection, mice were fasted for 12 h and anesthetized by intraperitoneal injection of 1% sodium pentobarbital (50 mg/kg). Peripheral blood was collected from the orbital venous plexus. One portion was centrifuged to obtain serum and stored at −80°C for ELISA, whereas the remaining portion was used for RNA extraction and reverse transcription into cDNA, which was stored at −20°C until qRT‐PCR analysis. After blood collection, the mice were transcardially perfused with iced PBS. Brain tissues were then rapidly dissected on ice and processed for different assays: some tissues were preserved for qRT‐PCR, some were homogenized in RIPA lysis buffer containing PMSF for Western blot analysis, and others were fixed in 4% PFA, dehydrated, paraffin‐embedded, and sectioned at 8 μm for immunofluorescence staining.

### 2.12 qRT‐PCR Detection

Total RNA was extracted from peripheral blood and brain tissue using the RNeasy Mini Kit according to the manufacturer's instructions. The purity and concentration of RNA were detected using a NanoDrop 2000 spectrophotometer (Thermo Fisher Scientific, USA), and an A260/A280 ratio between 1.8 and 2.0 was considered qualified. One microgram of total RNA was used to remove genomic DNA contamination using the PrimeScript RT Reagent Kit with gDNA Eraser, followed by reverse transcription to synthesize complementary DNA (cDNA).

qRT‐PCR was conducted on a StepOnePlus system (Bio Rad, USA) with TB Green Premix Ex Taq II reagents under standard thermal cycling conditions, followed by melting curve analysis for specificity validation. GAPDH served as the internal control, and target gene expression was calculated via the 2^−ΔΔCt^ method. Primer sequences are listed in Table 1.

**TABLE 1:** Primer sequences for qRT‐PCR.

Gene	Forward primer (5′ → 3′)	Reverse primer (5′ → 3′)
S1PR5	TGTGTAGAGAACGATAACCTTGAAGTC	CAGGAGAGCGGCTATTGGAAATC
GAPDH	TGGAGAAACCTGCCAAGTATGA	TGGAAGAATGGGAGTTGCTGT

### 2.13 Western Blot (WB) Detection

Hippocampal tissues were lysed in RIPA buffer containing 1% PMSF and 1% phosphatase inhibitor cocktail (Beyotime, China), homogenized on ice, and centrifuged at 12,000 × g for 20 min at 4°C. Protein concentrations were determined using a BCA protein assay kit (Beyotime, China). Equal amounts of protein (50 μg) were separated by 10% SDS‐PAGE and transferred onto PVDF membranes (Millipore, USA). The membranes were blocked with 5% non‐fat milk in TBST and then incubated overnight at 4°C with primary antibodies against S1PR5, RhoA, ROCK1, ROCK2, phospho‐NF‐κB p65, NF‐κB p65, Claudin‐5, and ZO‐1 (all 1:1000), as well as GAPDH (1:5000). After washing, the membranes were incubated with the corresponding HRP‐conjugated secondary antibody (1:5000). Protein bands were visualized using an enhanced chemiluminescence (ECL) detection kit (GK10008, GlpBio, China), and images were acquired using a ChemiDoc XRS+ imaging system (Bio‐Rad, USA). Band intensities were quantified using ImageJ software (version 2.16.0, USA).

### 2.14 Immunofluorescence (IF) Staining

Paraffin sections were dewaxed with xylene and hydrated with graded ethanol (both Tianjinfengchuan, China). Antigen retrieval was performed by boiling the sections in 0.01 M citrate buffer (pH 6.0, Solaibio, China) for 30 min. After washing with PBS (HyClone), sections were blocked with PBS containing 5% donkey serum (Solaibio, China) at room temperature for 1 h, and then incubated separately with primary antibody (S1PR5: 1:100 dilution; Iba1: 1:200 dilution; CD31: 1:200 dilution; Claudin‐5: 1:200 dilution; NeuN: 1:400 dilution) at 4°C overnight. The next day, the sections were incubated with Alexa Fluor 488/594 secondary antibodies (1:1000 dilution, Invitrogen), stained with DAPI (1:1000 dilution, Abcam), and mounted with anti‐fade medium (Vector Laboratories). Images were acquired at 400× magnification using a Biotek Live‐Cell Imaging Microscope (Biotek), and fluorescence intensities were quantified with ImageJ 2.16.0 (USA).

### 2.15 ELISA Assay

The concentrations of TNF‐α, IL‐1β, IL‐6, IL‐10, S1PR5 in mouse serum and brain tissue were detected using commercial ELISA kits described above, strictly following the manufacturer's instructions.

### 2.16 Detection of BBB Permeability by EB Assay

Two hours before sacrifice, mice were injected with 2% EB solution (5 mL/kg) via the tail vein. After 2 h of EB circulation, mice were anesthetized and perfused transcardially with 0.9% NS until the perfusate became clear (to remove residual EB in blood vessels). Brains were harvested, weighed, and homogenized in formamide (Sigma‐Aldrich, USA) at a ratio of 500 μL per 50 mg of tissue. Following overnight incubation at 60°C, samples were centrifuged at 12000 × g for 30 min at 4°C. Absorbance at 620 nm was measured with a microplate reader. BBB permeability was quantified as μg EB per g brain tissue.

### 2.17 AAV‐Mediated S1PR5 Overexpression in Mice

For in vivo overexpression of S1PR5, mice received stereotaxic injection of AAV‐S1PR5 into the hippocampus. Mice injected with AAV‐control were used as controls. Briefly, mice were anesthetized and placed in a stereotaxic frame. AAV‐S1PR5 or AAV‐control was bilaterally injected into the hippocampus according to the following coordinates relative to bregma: AP = −1.5 mm, ML = 1.8 mm, DV = −2.3 mm. The mice were allowed to recover and were used for subsequent experiments after 2 weeks of viral expression. S1PR5 overexpression was verified by immunofluorescence staining.

### 2.18 Behavioral Assessment of Epileptic Seizures

Seizure severity was evaluated according to the Racine scale after KA administration [17]. Behavioral seizures were graded as follows: Stage 0, no obvious behavioral change; Stage 1, facial automatisms and mouth/facial movements; Stage 2, head nodding; Stage 3, unilateral forelimb clonus; Stage 4, bilateral forelimb clonus with rearing; and Stage 5, rearing and falling. Seizures with a Racine score of ≥ 3 were defined as high‐grade seizures. Seizure duration was recorded in seconds. The latency to seizure onset was defined as the time from drug administration to the first observed seizure. In addition, the number of seizures on Day 1 and Day 7 was recorded, and the duration of high‐grade seizures on Day 7 was further quantified. Behavioral scoring was performed independently by two investigators blinded to the group allocation.

### 2.19 Statistical Analysis

All experimental data were expressed as ‘mean ± standard deviation (mean ± SD)’. Statistical analysis was performed using the GraphPad Prism 8.0 software (GraphPad Software, San Diego, CA, USA). One‐way analysis of variance (ANOVA) was used for comparisons among multiple groups, and Tukey's post hoc test was used for pairwise comparisons between groups. A *p* value < 0.05 was considered statistically significant.

## 3 Results

### 3.1 S1PR5 Is Significantly Downregulated in Peripheral Blood of Epilepsy Patients

#### 3.1.1 Screening and Functional Localization of Epilepsy Differentially Expressed Genes (DEGs)

This study aimed to identify novel therapeutic targets and intervention strategies for epilepsy by integrating multi‐omics and machine learning approaches. Analysis of the GSE143272 dataset identified 767 differentially expressed genes (DEGs) and functional enrichment analysis revealed that these genes were significantly associated with immune and inflammatory response pathways (Figure 1a–e).

**FIGURE 1:**
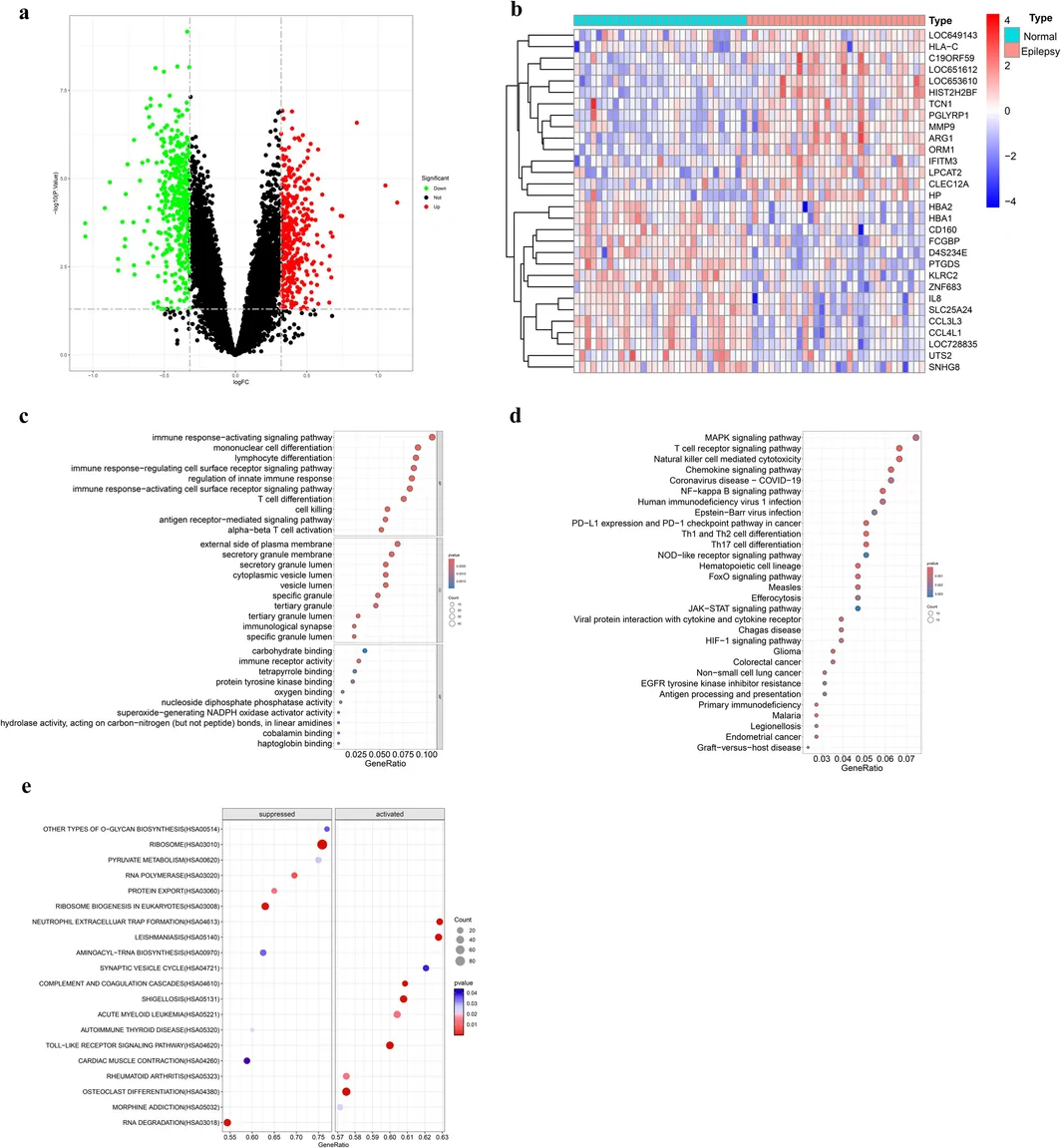
Identification and functional enrichment analysis of differentially expressed genes in epilepsy. (a) Volcano plot showing the distribution of DEGs between the epilepsy group and the control group. Green and red dots represent significantly downregulated and upregulated genes, respectively (screening criteria: *p*‐value < 0.05 and |log_2_FC| > 1); (b) Heatmap of DEGs. The abscissa represents samples (Normal/Epilepsy), and the ordinate represents DEGs. The color indicates gene expression levels (red for high expression, blue for low expression), with clear distinction between the two groups of samples; (c) Gene Set Enrichment Analysis (GSEA) showing the enrichment trend of ‘inflammation‐related pathways’ in the epilepsy group; (d) GSEA showing the enrichment trend of ‘immune cell activation pathways’ in the epilepsy group; (e) Bubble plot of Kyoto Encyclopedia of Genes and Genomes (KEGG) pathway enrichment analysis, showing the enrichment of DEGs in inflammation‐related pathways.

#### 3.1.2 Machine Learning Screening, Validation, and Target Gene Identification of Core Feature Genes

WGCNA identified the turquoise module as the module most strongly associated with the epilepsy phenotype (*p* = 2.2 × 10^−40^) (Figure 2a). Intersection analysis between the turquoise module genes and DEGs yielded 162 key differentially co‐expressed genes (Figure 2b), which were further subjected to PPI network construction (Figure 2c). To identify robust feature genes, LASSO, RF, and SVM analyses were performed (Figure 2d–f). Intersection of the genes identified by the three algorithms resulted in six core feature genes: S1PR5, ARG1, CXCL1, MMP9, MAPK14, and CD160 (Figure 2g). Among them, S1PR5 was significantly downregulated in epilepsy samples (Figure 2h) and showed good diagnostic value, with an AUC of 0.809 (Figure 2i). Based on its differential expression, diagnostic performance, predicted drug‐target interaction, and prior evidence suggesting that S1PR5 may be involved in antiepileptic drug resistance and blood–brain barrier protection, S1PR5 was selected for subsequent validation [16, 18].

**FIGURE 2:**
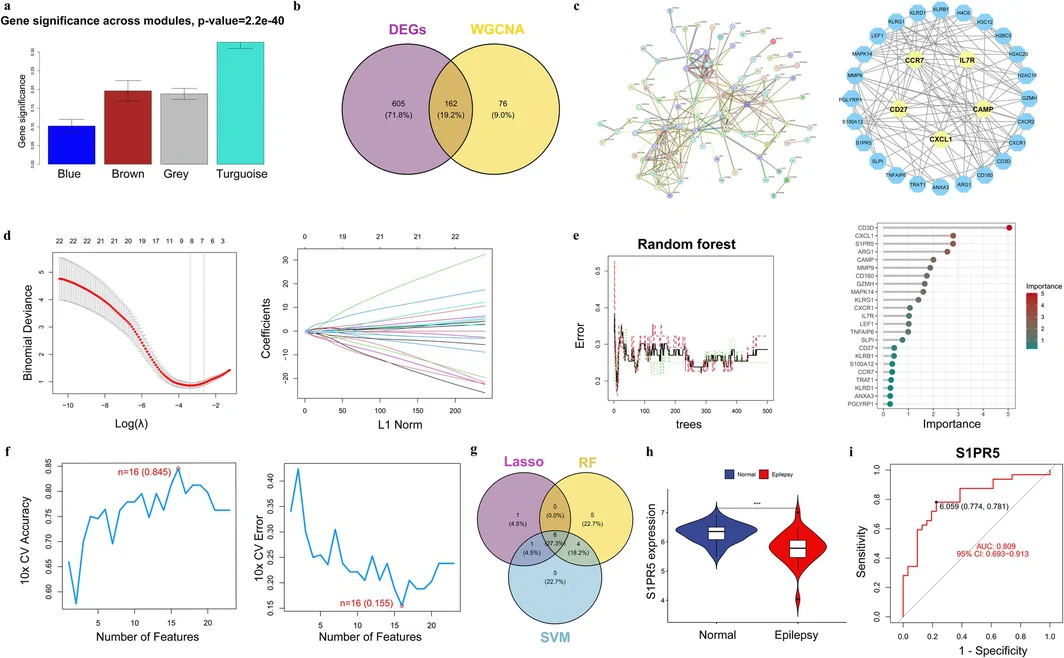
Screening and validation of the core epilepsy‐related feature gene S1PR5. (a) Box plot showing the distribution of gene significance in different modules from WGCNA, with the turquoise module exhibiting the highest correlation with epilepsy phenotype (*p*‐value = 2.2e‐40); (b) Venn diagram showing the intersection between DEGs and genes in the turquoise module (162 key differentially co‐expressed genes); (c) Comparison diagram of PPI networks: Left, full network; right, core node gene network; (d) Coefficient trajectory plot (upper) and cross‐validation mean squared error curve (lower) from LASSO regression analysis, used for feature gene screening; (e) Feature gene importance ranking (left) and distribution (right) in the Random Forest model; (f) Cross‐validation accuracy (left) and mean squared error (right) curves of LASSO regression, with the optimal number of features *m* = 16; (g) Venn diagram showing the intersection of feature genes screened by LASSO, Random Forest (RF), and SVM (6 core feature genes); (h) Bean plot showing the expression of S1PR5 between the normal group and the epilepsy group. *** indicates extremely significant difference (*p* < 0.001); (i) ROC curve of S1PR5, with AUC = 0.809, indicating good diagnostic efficacy.

### 3.2 Overexpression of S1PR5 Inhibited Epileptic Seizures in KA‐Treated Mice

To clarify the functional role of S1PR5 in epileptic pathogenesis, adeno‐associated virus (AAV)‐mediated S1PR5 overexpression was performed in mouse hippocampi before KA administration. The efficiency of S1PR5 overexpression was verified by IF, showing markedly elevated S1PR5 protein levels in the KA + S1PR5 OE group relative to the KA + AAVctrl group (**p < 0.01; Figure 3a). Immunofluorescence staining demonstrated that S1PR5 overexpression significantly restrained the number of FJC‐labeled neurons (*p < 0.05; Figure 3b) and Iba1‐labeled microglia (*p < 0.05; Figure 3c) compared with the KA + AAVctrl group. Meanwhile, behavioral assessments revealed that S1PR5 overexpression led to prolonged latent phase of seizure, low frequent seizures, and shorter durations of severe seizures (**p < 0.01 or *p < 0.05; Figure 3d). These data demonstrate that S1PR5 plays a protective role in epilepsy, and its downregulation contributes to neuronal damage and seizure susceptibility.

**FIGURE 3:**
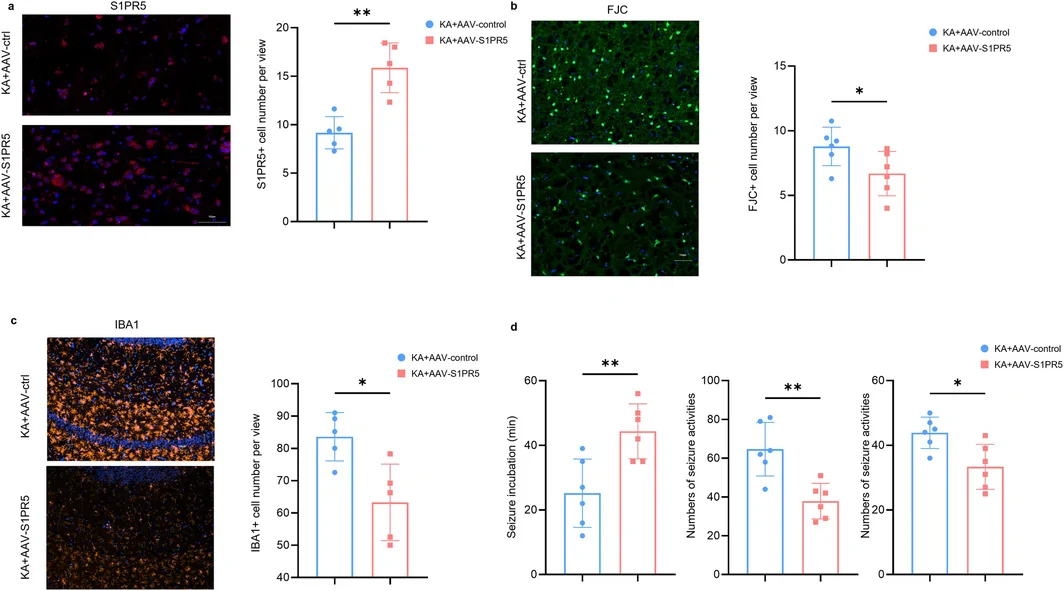
S1PR5, NeuN, and Iba1 immunofluorescence staining and seizure activity analysis in KA + AAV‐control and KA + AAV‐S1PR5 groups. (a) Results of S1PR5 immunofluorescence staining and comparison of S1PR5^+^ cell number in brain between the KA + AAV‐control group and the KA+ AAV‐S1PR5 group; (b) Results of NeuN (neuronal marker) immunofluorescence staining and comparison of NeuN neurons number in brain between the KA + AAV‐control group and the KA+ AAV‐S1PR5 group; (c) Results of Iba1 (microglial marker) immunofluorescence staining and comparison of microglial cells in brain between the KA + AAV‐control group and the KA+ AAV‐S1PR5 group; (d) Comparison of seizure incubation period (left) and Number of seizure activities in the acute phase (Day 1, middle) and chronic phase (Day 7, right) between the KA + AAV‐control group and the KA+ AAV‐S1PR5 group. Data were shown as mean ± SD, unpaired Student's t‐test, *p < 0.05, **p < 0.01, ***p < 0.001, ns = no statistical significance.

### 3.3 Molecular Docking Validation of Fingolimod as a Potential S1PR5‐Targeting Anti‐Epileptic Agent

To explore the therapeutic feasibility of targeting S1PR5, we performed molecular docking simulations. The results showed that Fingolimod, a clinically approved drug, could stably bind to the binding pocket (CurPocket ID C3) of the S1PR5 protein with high binding affinity (Vina score = −7.1, Figure 4). This key finding suggests that Fingolimod, as a known sphingosine‐1‐phosphate receptor (S1PR) modulator, may exert its previously unrecognized anti‐epileptic effects by directly acting on S1PR5, a novel target identified in this study.

**FIGURE 4:**
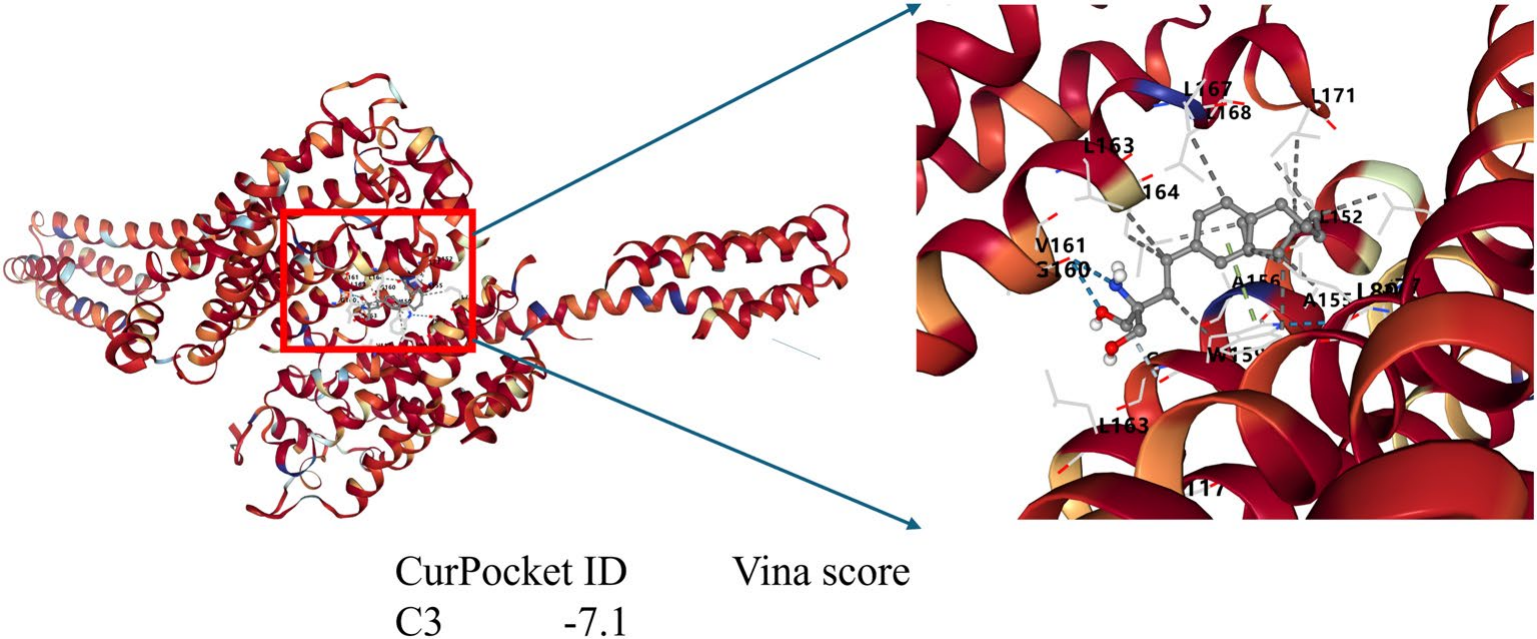
Molecular docking result diagram showing the stable binding of Fingolimod to the binding pocket (CurPocket ID C3) of S1PR5 (Vina score = −7.1).

### 3.4 Fingolimod Can Reverse the Downregulation of S1PR5 During Epileptic Progression

To examine whether Fingolimod regulates S1PR5 in epileptic conditions, we measured S1PR5 expression in control, KA, and KA + Fingolimod groups; we performed transcriptional analyses in the mouse hippocampus. Compared with the control group, the KA‐induced epilepsy model group exhibited significantly lower mRNA levels of S1PR5 in both brain tissue and peripheral blood (*p < 0.05 or **p < 0.01; Figure 5a–c). Following Fingolimod intervention, S1PR5 transcription was significantly restored, approaching levels comparable to those in the control group (*p < 0.05; Figure 5a–c).

**FIGURE 5:**
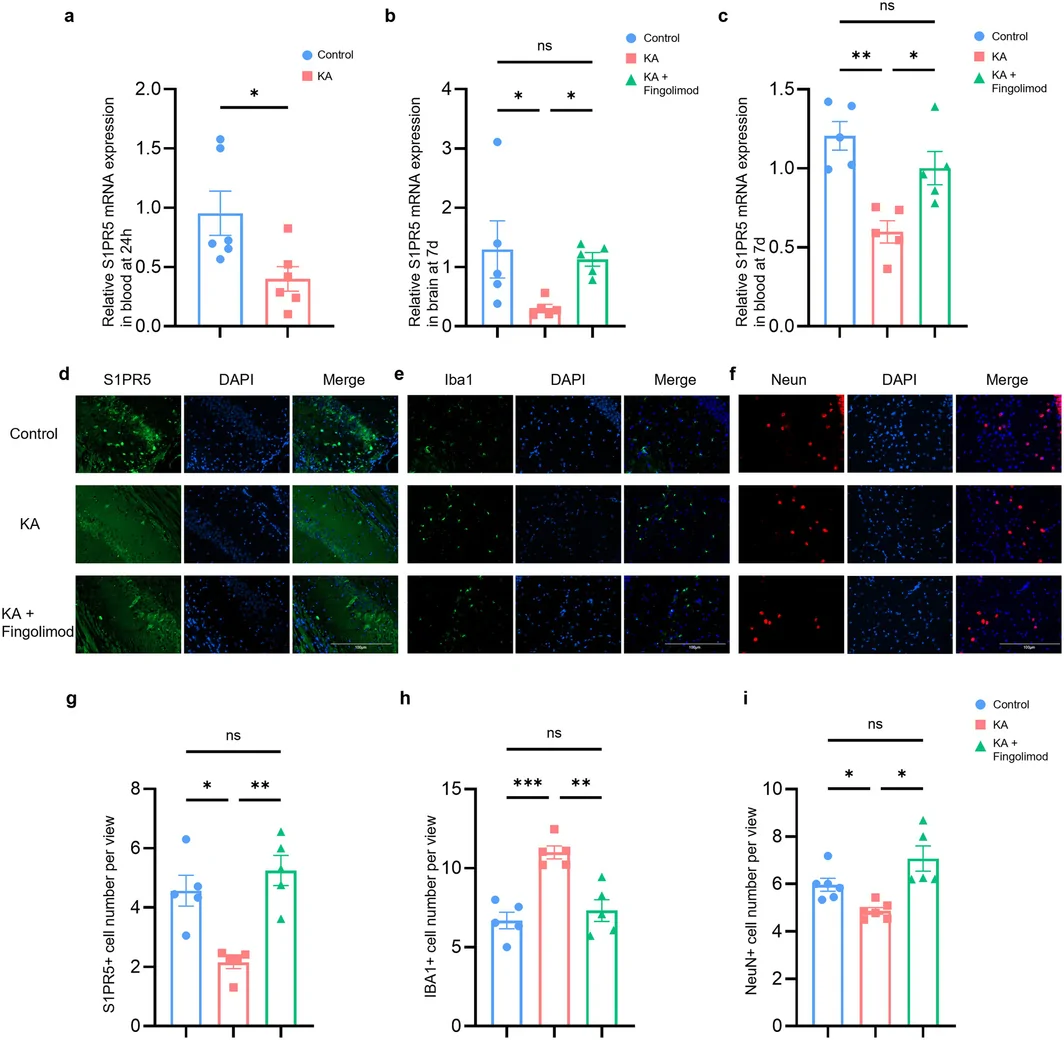
Analysis of S1PR5 expression, microglial activation, and neuronal changes in KA‐induced epileptic mice. (a) Comparison of relative mRNA expression levels of S1PR5 between the control group and theKA‐induced epilepsy model group; (b) Comparison of S1PR5 mRNA expression in brain tissue among the control group, KA group, KA + Fingolimod group; (c) Comparison of S1PR5 mRNA expression in peripheral blood among the control group, KA group, and KA + Fingolimod group; (d) Results of S1PR5 immunofluorescence staining; (e) Results of Iba1 (microglial marker) immunofluorescence staining; (f) Results of NeuN (neuronal marker) immunofluorescence staining; (g) Statistical analysis of the number of S1PR5 cells; (h) Statistical analysis of the number of Iba1 microglia; (i) Statistical analysis of the number of NeuN neurons. Blue circles represent the control group, red squares represent the KA group, and green triangles represent the KA + Fingolimod group. Data were shown as mean ± SD, unpaired Student's t‐test for a; one‐way ANOVA for b–i, *p < 0.05, **p < 0.01, ***p < 0.001, ns = no statistical significance.

At protein and cellular levels, S1PR5 immunofluorescence results showed that S1PR5‐positive cells (green fluorescence) were abundant in the brain tissue of the control group, with nuclei counterstained by DAPI (blue fluorescence). In the KA group, the number of S1PR5‐positive cells was markedly reduced, whereas Fingolimod treatment significantly increased the number of S1PR5‐positive cells compared with the KA group (*p < 0.05 or **p < 0.01; Figure 5d,g). Iba1 immunofluorescence staining showed a significant increase in the number of Iba1‐positive microglia in the KA group, which was attenuated in the KA + Fingolimod group (***p < 0.001 or **p < 0.01; Figure 5e,h). NeuN immunofluorescence results revealed a significant reduction in the number of NeuN‐positive neurons in the KA group, whereas this reduction was partially reversed by Fingolimod treatment (*p < 0.05; Figure 5f,i).

### 3.5 Fingolimod Maintains Blood–Brain Barrier Integrity

EB leakage assay was used to evaluate BBB integrity in epileptic mice. The KA group showed significantly increased EB absorbance in brain tissue compared with the control group (**p < 0.01), indicating BBB disruption. Fingolimod treatment markedly reduced EB absorbance (***p < 0.001), restoring it to a level comparable to that of the control group (Figure 6a). Immunofluorescence staining further showed that Claudin‐5 fluorescence intensity was markedly decreased in the KA group compared with the control group (*p < 0.05 or **p < 0.01; Figure 6b,c), whereas Fingolimod treatment significantly reversed this reduction (**p < 0.05), indicating restoration of BBB tight junction integrity.

**FIGURE 6:**
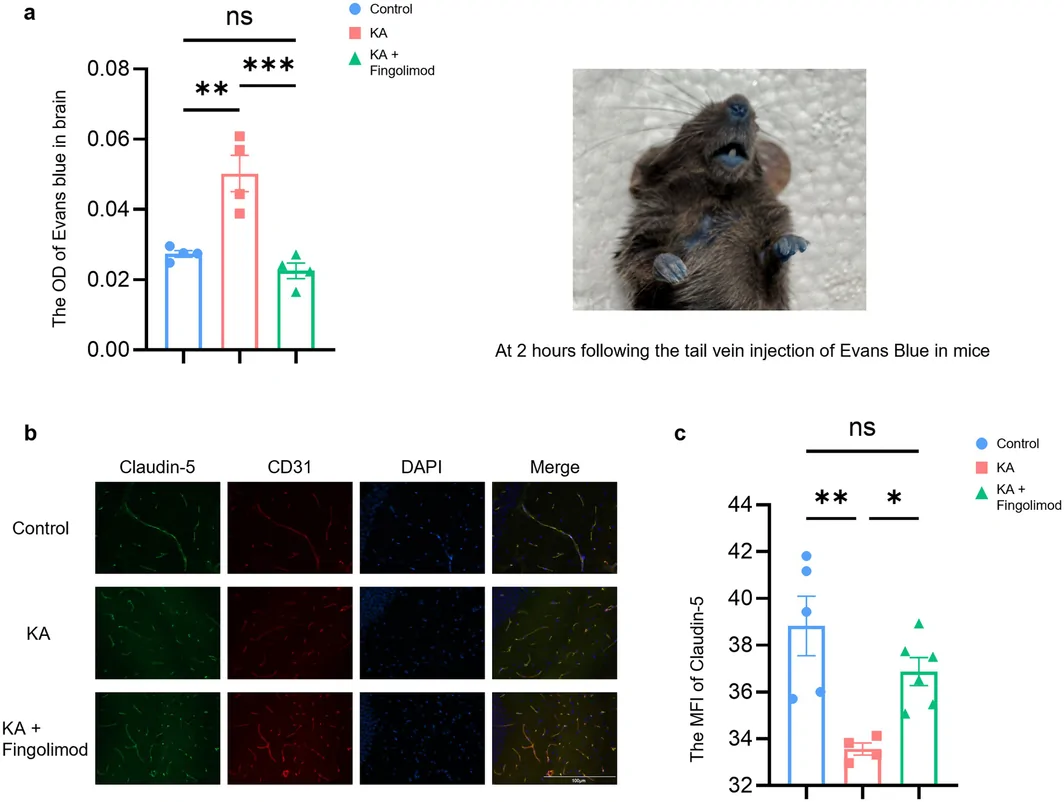
Analysis of blood–brain barrier (BBB) integrity by Evans Blue (EB) leakage assay and claudin‐5/CD31 immunofluorescence. (a) Quantitative analysis of EB absorbance (OD value) in the brain and schematic diagram of experimental mice among different treatment groups (Control, KA, KA + Fingolimod); (b) Results of claudin‐5 and CD31 immunofluorescence staining in brain; (c) Statistical analysis of the intensity of claudin‐5. Blue circles represent the control group, red squares represent the KA group, and green triangles represent the KA + Fingolimod group. Data were shown as mean ± SD, one‐way ANOVA, *p < 0.05, **p < 0.01, ns = no statistical significance.

### 3.6 Fingolimod Attenuates Neuroinflammation

To investigate the relationship between S1PR5 expression and inflammatory responses, as well as the effects of Fingolimod treatment, S1PR5 and cytokine levels were measured in brain tissue and serum. S1PR5 levels were significantly reduced in the KA group (*p < 0.05 or **p < 0.01; Figure 7a,c), whereas Fingolimod restored S1PR5 expression to levels comparable to those in the control group. Compared with the control group, the KA group showed significantly increased levels of IL‐6, IL‐1β, and TNF‐α, but a decreased level of IL‐10 (*p < 0.05, **p < 0.01, or ***p < 0.001; Figure 7b,d). These changes were significantly reversed by Fingolimod treatment.

**FIGURE 7:**
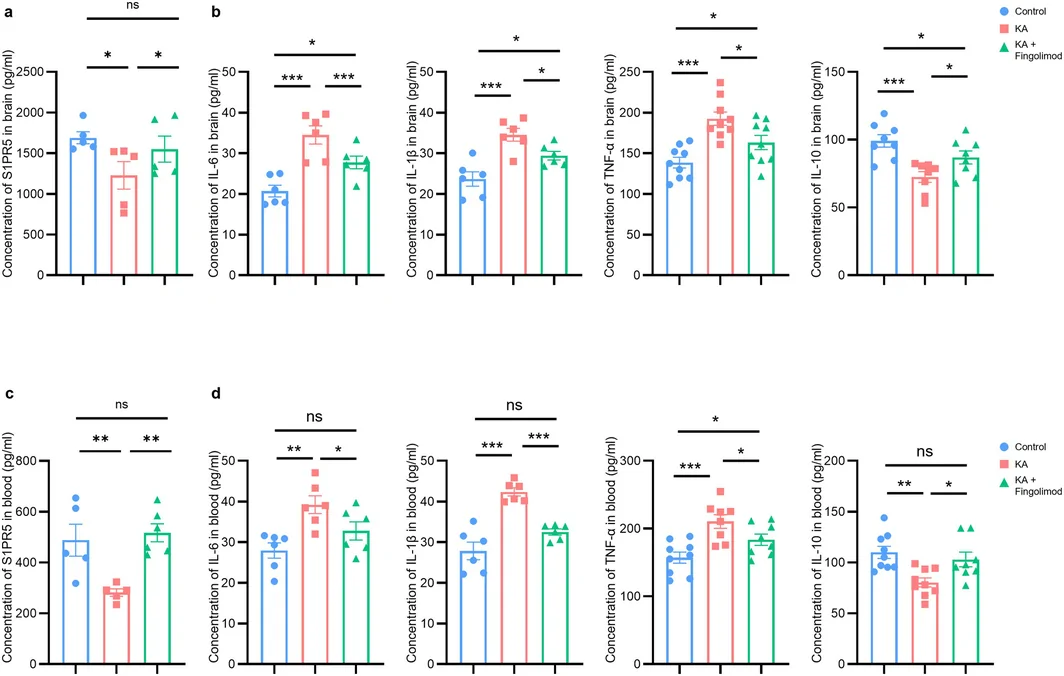
Enzyme‐linked immunosorbent assay (ELISA) detection results of S1PR5and cytokinesin tissues and serum. (a) The concentration comparison of S1PR5 in brain. (b) The concentration comparison of cytokines (left to right: IL‐6, IL‐1β, TNF‐α, IL‐10) in brain. (c) The concentration comparison of S1PR5 in serum. (d) The concentration comparison of cytokines (left to right: IL‐6, IL‐1β, TNF‐α, IL‐10) in serum. Blue circles represent the control group, red squares represent the KA group, and green triangles represent the KA + Fingolimod group. Data were shown as mean ± SD, one‐way ANOVA, *p < 0.05, **p < 0.01, ***p < 0.001, ns = no statistical significance.

### 3.7 Fingolimod Inhibits Abnormal Activation of the Rho/ROCK/NF‐κB Pathway

In view of the confirmation that fingolimod exerts a neuroprotective effect in epilepsy by restoring the function of S1PR5, we further investigate the molecular mechanism underlying the functional action of fingolimod. Previous research has confirmed that S1PRs regulate disease progression across multiple pathologies by modulating the Rho/ROCK/NF‐κB signaling pathway [19, 20]. Thus we hypothesized that Fingolimod may alleviate epileptic pathology by suppressing the Rho/ROCK/NF‐κB pathway in an S1PR5‐dependent manner. Western blot analysis showed that S1PR5 expression was significantly lower in the KA group than in the control group, while it was restored in the KA+ Fingolimod group (****p < 0.0001; Figure 8a), indicating that Fingolimod upregulates S1PR5. Regarding the molecules of the Rho/ROCK/NF‐κB pathway, the KA group exhibited significantly elevated Rho, RhoA, ROCK1, ROCK2, and p‐p65 levels (****p < 0.0001, ***p < 0.001, or *p < 0.05; Figure 8c–h), whereas total p65 remained unchanged. Fingolimod significantly suppressed these pathway molecules, suggesting that Fingolimod can inhibit the activation of the Rho/ROCK/NF‐κB pathway. Meanwhile, ZO‐1 was decreased in the KA group (****p < 0.0001) and restored by Fingolimod treatment (***p < 0.001; Figure 8b).

**FIGURE 8:**
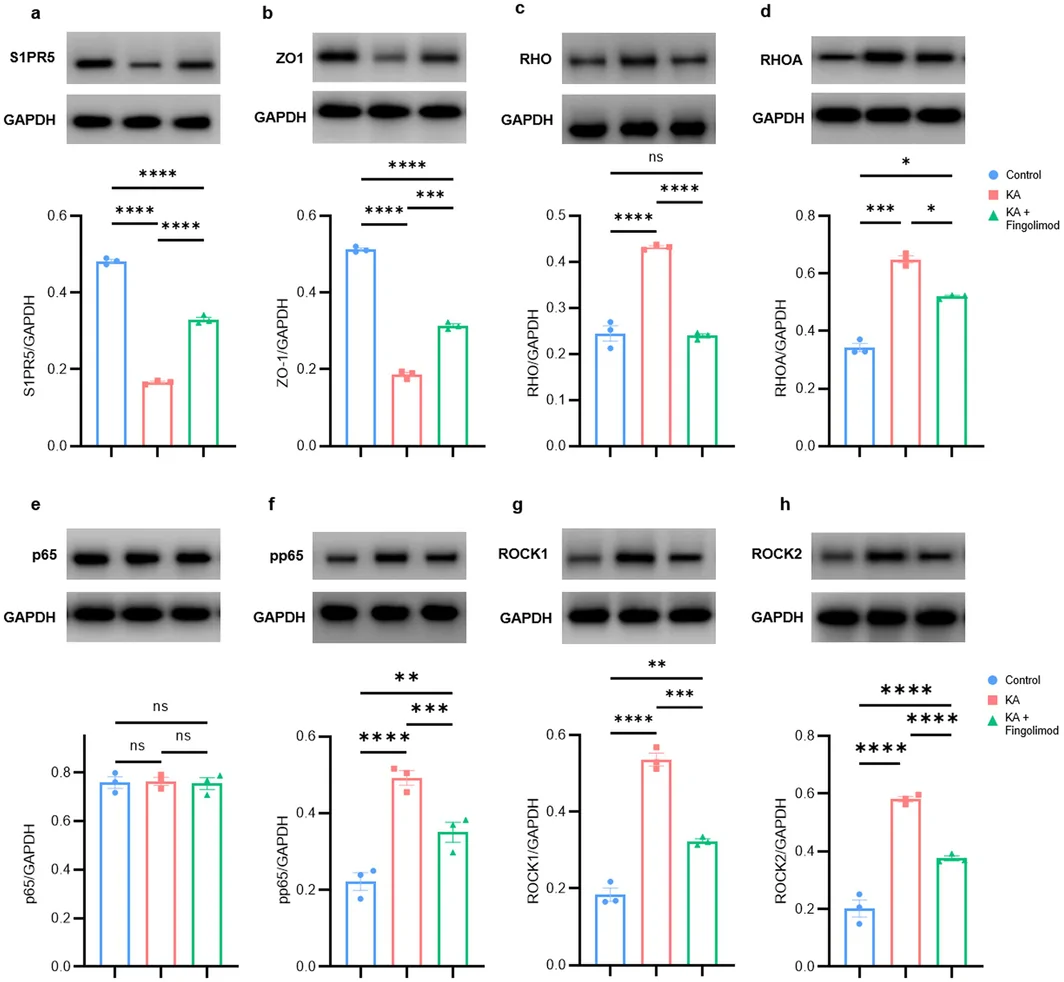
Western blot (WB) detection of Rho/ROCK/NF‐κB pathway and blood–brain barrier (BBB)‐associated proteins. Representative Western blot images and densitometric analysis of S1PR5 (a), ZO‐1 (b), RHO (c), RHOA (d), p65 (e), pp65 (f), ROCK1 (g), and ROCK2 (h) in the Control, KA, and KA + Fingolimod groups. Representative blots are shown above each graph, and the quantified protein levels normalized to GAPDH are shown below; from left to right, the groups are Control, KA, and KA + Fingolimod. Blue circles represent the control group, red squares represent the KA group, and green triangles represent the KA + Fingolimod group. Data were shown as mean ± SD, one‐way ANOVA for (b–i), *p < 0.05, **p < 0.01, ***p < 0.001, ****p < 0.0001, ns = no significant difference.

### 3.8 Improvement Effect of Fingolimod on the Behaviors of Epileptic Model Mice

Behavioral tests were performed to assess the antiepileptic effects of Fingolimod. Compared with the KA group, the KA + Fingolimod group showed a significantly prolonged seizure latency, indicating that Fingolimod effectively delayed seizure onset (*p < 0.05; Figure 9a). In addition, the number of acute seizures on Day 1 (**p < 0.01; Figure 9b), the total number of seizures on Day 7 (*p < 0.05; Figure 9c), and the duration of high‐grade seizures (Racine score ≥ 3) during the chronic phase on Day 7 (**p < 0.01; Figure 9d) were all significantly reduced by Fingolimod treatment. These results suggest that Fingolimod attenuated both seizure frequency and severity in epileptic mice.

**FIGURE 9:**
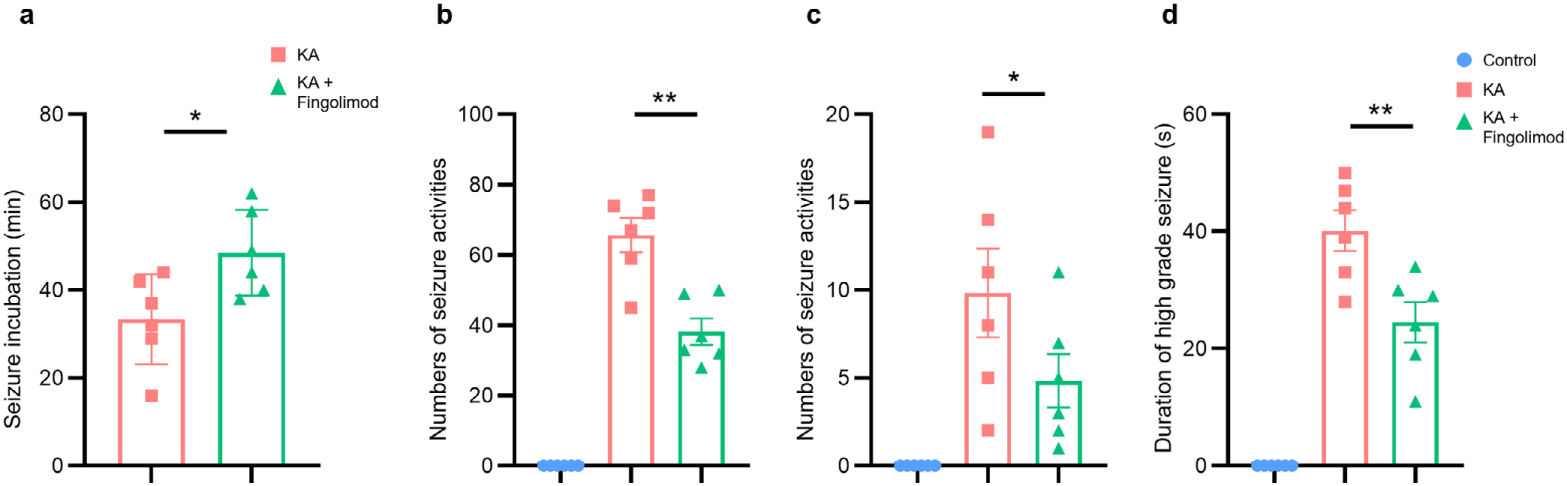
Behavioral effects of Fingolimod on kainic acid (KA)‐induced epileptic model mice. (a) Seizure incubation period. (b) Number of seizure activities in the acute phase (Day 1). (c) Number of seizure activities in the chronic phase (Day 7). (d) Duration of high‐grade seizures in the chronic phase (Day 7). Data were shown as mean ± SD, unpaired Student's *t*‐test, *p < 0.05, **p < 0.01, ***p < 0.001, ns = no statistical significance.

## 4 Discussion

This study demonstrates that Fingolimod markedly attenuates neuroinflammation, preserves blood–brain barrier (BBB) integrity, and improves seizure‐related behavioral abnormalities in kainic acid (KA)‐induced epilepsy. By integrating bioinformatics analysis, molecular docking, and experimental validation, we showed that S1PR5 was significantly downregulated in epilepsy and that Fingolimod restored its expression. This restoration was accompanied by inhibition of the Rho/ROCK/NF‐κB pathway, reduction of inflammatory cytokine release, and improvement of BBB integrity. Collectively, these findings support an important role for S1PR5‐associated signaling in the pathological process of epilepsy and identify S1PR5 as a promising molecular candidate underlying the therapeutic effects of Fingolimod.

First, this study found that S1PR5 expression was significantly reduced in both patients with epilepsy and experimental animals, strongly suggesting that S1PR5 is involved in epileptic pathogenesis. This finding is consistent with the emerging understanding of sphingosine‐1‐phosphate receptors (S1PRs) in neurological disorders. S1PR5 is expressed in the central nervous system, particularly in oligodendrocytes and certain glial cell populations, and has been implicated in the regulation of cell migration, survival, and inflammatory responses [21]. Previous studies have also shown that S1PR5 contributes to endothelial stability and BBB maintenance [22]. Therefore, the downregulation of S1PR5 observed in epilepsy may be closely associated with impaired barrier homeostasis, enhanced inflammatory activation, and aggravated tissue injury.

Fingolimod is a well‐established S1PR modulator with both immunomodulatory and neuroprotective properties. It can cross the BBB and regulate S1PR‐related signaling in immune cells as well as in cells within the central nervous system [23]. Previous studies have shown that Fingolimod can inhibit the aberrant activation of S1PR‐related signaling, restore receptor homeostasis, and alleviate inflammation‐related injury in multiple disease settings [24]. In the present study, Fingolimod restored the reduced expression of S1PR5 in epileptic mice, and molecular docking analysis supported a favorable interaction between Fingolimod and S1PR5. Although direct receptor‐binding and receptor‐specific functional assays are still required for definitive confirmation, our findings strongly support the view that S1PR5 is closely involved in the therapeutic actions of Fingolimod in epilepsy.

Another important finding is that the protective effects of Fingolimod were accompanied by suppression of the Rho/ROCK/NF‐κB signaling pathway. The Rho/ROCK pathway regulates cytoskeletal remodeling, endothelial permeability, and inflammatory activation, whereas NF‐κB is a central mediator of inflammatory amplification [25, 26]. Previous studies have shown that S1PR5 can couple with G12/13 proteins to regulate Rho activity [27, 28], suggesting a mechanistic link between S1PR5‐related signaling and the Rho/ROCK pathway. Consistently, KA‐induced epilepsy in our study was characterized by reduced S1PR5 expression and activation of the Rho/ROCK/NF‐κB pathway, whereas Fingolimod restored S1PR5 expression and suppressed pathway activation. These findings support a functional association between S1PR5‐related signaling and the Rho/ROCK/NF‐κB axis in epilepsy.

BBB dysfunction is increasingly recognized as a key contributor to epileptogenesis and seizure recurrence. In the present study, KA treatment increased Evans Blue leakage, reduced ZO‐1 expression, and decreased Claudin‐5 immunofluorescence, indicating BBB disruption. Fingolimod significantly reduced BBB permeability and restored both ZO‐1 and Claudin‐5 expression, indicating a protective effect on BBB structure and function. This finding echoes the role of Fingolimod in protecting the BBB in multiple sclerosis [29]. Together with the restoration of S1PR5 expression and inhibition of Rho/ROCK/NF‐κB signaling, these findings support the involvement of S1PR5‐associated signaling in BBB protection in epilepsy.

Neuroinflammation is another central process in epilepsy progression. Activated microglia and excessive inflammatory cytokine release can enhance neuronal excitability and further disrupt the BBB, thereby forming a self‐amplifying pathological cycle [30, 31, 32]. In our study, the KA group showed increased Iba1‐positive microglia, elevated IL‐6, IL‐1β, and TNF‐α levels, and reduced IL‐10 levels. Fingolimod significantly reversed these changes, indicating a robust anti‐inflammatory effect.

At the behavioral level, Fingolimod significantly prolonged seizure latency and reduced seizure frequency and severity in KA‐induced epileptic mice, indicating a clear therapeutic benefit in vivo. These behavioral improvements were consistent with the molecular, inflammatory, and BBB‐protective changes observed in this study, supporting the overall antiepileptic efficacy of Fingolimod. Given its dual actions on neuroinflammation and BBB dysfunction, Fingolimod may represent a promising therapeutic candidate for epilepsy, particularly for patients with prominent inflammatory activation and barrier impairment.

Several limitations should be acknowledged. First, although molecular docking and expression analyses support the involvement of S1PR5, direct target‐binding and receptor‐specific functional studies are still needed to establish causality. Second, the precise downstream mechanisms linking S1PR5 to Rho/ROCK/NF‐κB signaling require further clarification. Third, these findings were obtained in a KA‐induced model, and their applicability to other epilepsy models and clinical settings remains to be determined. Nevertheless, this study provides evidence that S1PR5 is closely associated with epilepsy‐related neuroinflammation and BBB dysfunction, and highlights S1PR5 as a promising therapeutic target in epilepsy.

## 5 Conclusion

In summary, this study supports that S1PR5 is a potential key target involved in the pathogenesis and progression of epilepsy. Fingolimod may exert anti‐epileptic effects by restoring S1PR5 expression and inhibiting the Rho/ROCK/NF‐κB signaling pathway, thereby achieving the dual effects of BBB protection and neuroinflammation reduction. These findings not only improve our understanding of the biological role of S1PR5 in epilepsy but also provide bioinformatics and experimental evidence supporting the potential repurposing of Fingolimod for epilepsy treatment. Furthermore, this study provides a basis for future translational and clinical investigations targeting neuroinflammation‐ and BBB‐related mechanisms in epilepsy.

## Author Contributions

Z.Z. conceptualized and designed the study. C.Z., Y.D., and M.M. performed the most data analysis and interpretation. C.Z., W.‐N.J., participated in the data interpretation and visualization. L.X., C.Z., and Z.Z. wrote the initial draft. All authors have thoroughly reviewed the manuscript and have given their approval for the final version for publication.

## Funding

This work was supported by Tianjin Municipal Education Commission Scientific Research Project (no. 2023KJ116).

## Ethics Statement

All animal experiments were approved by the Tianjin Medical University General Hospital Animal Ethics Committee (approval no.: IRB2025‐DWFL‐662) and strictly conducted in accordance with the National Institutes of Health (NIH) Guide for the Care and Use of Laboratory Animals.

## Conflicts of Interest

The authors declare no conflicts of interest.

## Data Availability

The data that support the findings of this study are available from the corresponding author upon reasonable request.
